# Supporting deprescribing in hospitalised patients: formative usability testing of a computerised decision support tool

**DOI:** 10.1186/s12911-021-01484-z

**Published:** 2021-04-05

**Authors:** Melissa T. Baysari, Mai H. Duong, Patrick Hooper, Michaela Stockey-Bridge, Selvana Awad, Wu Yi Zheng, Sarah N. Hilmer

**Affiliations:** 1 Discipline of Biomedical Informatics and Digital Health, Faculty of Medicine and Health, Charles Perkins Centre, D17, The University of Sydney, Sydney, NSW 2006 Australia; 2 Kolling Institute of Medical Research, Faculty of Medicine and Health, University of Sydney and Royal North Shore Hospital, Sydney, Australia; 3 Departments of Clinical Pharmacology and Aged Care, Royal North Shore Hospital, Sydney, Australia; 4 eMR Connect Program, eHealth NSW, Sydney, Australia; 5 Clinical Engagement and Patient Safety, eHealth NSW, Sydney, Australia; 6 Black Dog Institute, Sydney, NSW Australia

**Keywords:** Computerised decision support, Deprescribing, Usability, Polypharmacy, Hospital

## Abstract

**Background:**

Despite growing evidence that deprescribing can improve clinical outcomes, quality of life and reduce the likelihood of adverse drug events, the practice is not widespread, particularly in hospital settings. Clinical risk assessment tools, like the Drug Burden Index (DBI), can help prioritise patients for medication review and prioritise medications to deprescribe, but are not integrated within routine care. The aim of this study was to conduct formative usability testing of a computerised decision support (CDS) tool, based on DBI, to identify modifications required to the tool prior to trialling in practice.

**Methods:**

Our CDS tool comprised a DBI MPage in the electronic medical record (clinical workspace) that facilitated review of a patient’s DBI and medication list, access to deprescribing resources, and the ability to deprescribe. Two rounds of scenario-based formative usability testing with think-aloud protocol were used. Seventeen end-users participated in the testing, including junior and senior doctors, and pharmacists.

**Results:**

Participants expressed positive views about the DBI CDS tool but testing revealed a number of clear areas for improvement. These primarily related to terminology used (i.e. what is a DBI and how is it calculated?), and consistency of functionality and display. A key finding was that users wanted the CDS tool to look and function in a similar way to other decision support tools in the electronic medical record. Modifications were made to the CDS tool in response to user feedback.

**Conclusion:**

Usability testing proved extremely useful for identifying components of our CDS tool that were confusing, difficult to locate or to understand. We recommend usability testing be adopted prior to implementation of any digital health intervention. We hope our revised CDS tool equips clinicians with the knowledge and confidence to consider discontinuation of inappropriate medications in routine care of hospitalised patients. In the next phase of our project, we plan to pilot test the tool in practice to evaluate its uptake and effectiveness in supporting deprescribing in routine hospital care.

**Supplementary Information:**

The online version contains supplementary material available at 10.1186/s12911-021-01484-z.

## Background

Deprescribing is the process of discontinuing a medication, supervised by clinicians, when the potential benefits of taking that medication no longer outweigh the potential harms to the patient [1, 2]. Despite the feasibility of deprescribing and the growing evidence that deprescribing can improve clinical outcomes, quality of life and reduce the likelihood of adverse drug events [1, 3, 4], the practice is not widespread, particularly in hospital settings.

Limited work has specifically focused on understanding the complexities surrounding deprescribing in hospitals, although this setting provides a safe context to trial deprescribing, with frequent access to healthcare professionals and close monitoring of patients. A recent study comprising interviews with geriatricians and pharmacists across four UK hospitals, identified a number of barriers to deprescribing in this context [5]. These included barriers related to professional roles (i.e. perceived scope of practice, lack of confidence), to the inpatient environment (i.e. limited information, acute nature of admission), and to attitudes and beliefs (e.g. attitudes towards medications, beliefs about risks) [5]. These barriers are consistent with those identified in a study conducted across six Australian hospitals, where many healthcare professionals did not consider deprescribing to be their responsibility, felt insecure and uncertain about deprescribing, and reported that it did not fit with the timeframe and workflow of an acute admission [6].

Clinical risk assessment tools can help prioritise patients for medication review and prioritise medications to deprescribe. One well validated and evidence-based tool is the Drug Burden Index (DBI). The DBI is a measure of a patient’s total exposure to medications with anticholinergic and sedative properties [7]. International pharmaco-epidemiological studies show that high DBI is associated with adverse outcomes in older people such as poor physical function, falls, frailty and death [8, 9]. In pre-clinical studies, high DBI has been shown to cause functional impairment and frailty, which is reversible on deprescribing [10]. DBI calculated without software has been used as an intervention [11] and an outcome [12] in clinical deprescribing studies. Stand-alone, web-based software to calculate and report on DBI has been used in clinical research as a risk assessment tool in the community [13, 14] and hospital setting [15]. Thus, easy access to a patient’s DBI during a hospital admission could assist in identifying at-risk patients in whom deprescribing may be appropriate. Studies have shown that when clinicians are presented with a patient’s DBI (i.e. a paper-based DBI report), they view this as useful [13, 16] and it affects deprescribing recommendations. However, no research to date has investigated the impact of a patient’s DBI integrated into the electronic medical record on deprescribing or patient outcomes.

With the rapid uptake of health information technology, new ways of supporting clinicians to deprescribe have now emerged in the hospital setting. Two recent literature reviews highlight the potential positive impact computerised decision support (CDS) can have on inappropriate medication use [17, 18]. There is also some evidence to suggest that CDS is cost effective [19–21], but further studies examining cost outcomes are needed. What these reviews do show is that poor user acceptance of CDS is a significant barrier to uptake and effectiveness of the tools, and authors have recommended that future work focus on improving the design of CDS, ensuring better integration into workflow [17, 18]. The recent international SENATOR trial of the STOPP/START prescribing criteria in older inpatients found poor uptake of the recommendations made by the CDS [22], despite good uptake of, and outcomes from, recommendations delivered by clinical staff in previous studies [23]. That is, only 15% of CDS recommendations were followed by clinicians in the SENATOR trial [22], compared to 84% when recommendations were delivered by clinical researchers in another randomized controlled trial [23].

There is now little doubt that designing technology that meets users’ needs and preferences, via a process of co-design with users, is necessary for successful implementation and uptake of health information technology [24]. This current study represented a final step in a co-design process with doctors, nurses and pharmacists to develop a CDS tool, based on DBI, to facilitate deprescribing in hospitalised patients. The aim of this study was to conduct formative usability testing of our CDS tool to identify well-designed aspects and modifications required before pilot testing the tool in practice.

## Method

### Design

Two rounds of scenario-based formative usability testing with think-aloud protocol were used. Usability testing, in particular, the think-aloud method, has been identified as one of the most effective ways to identify usability problems with digital health tools [25]. Think-aloud requires users to continuously verbalise their thoughts as they interact with a tool or system, allowing participants’ thought processes, feedback and emotional responses to be captured in real-time [26]. This technique has been used to evaluate CDS tools in other contexts [27–29].

Round 1 testing occurred in July 2019 and Round 2 in September 2019.

### Intervention

**Fig. 1:**
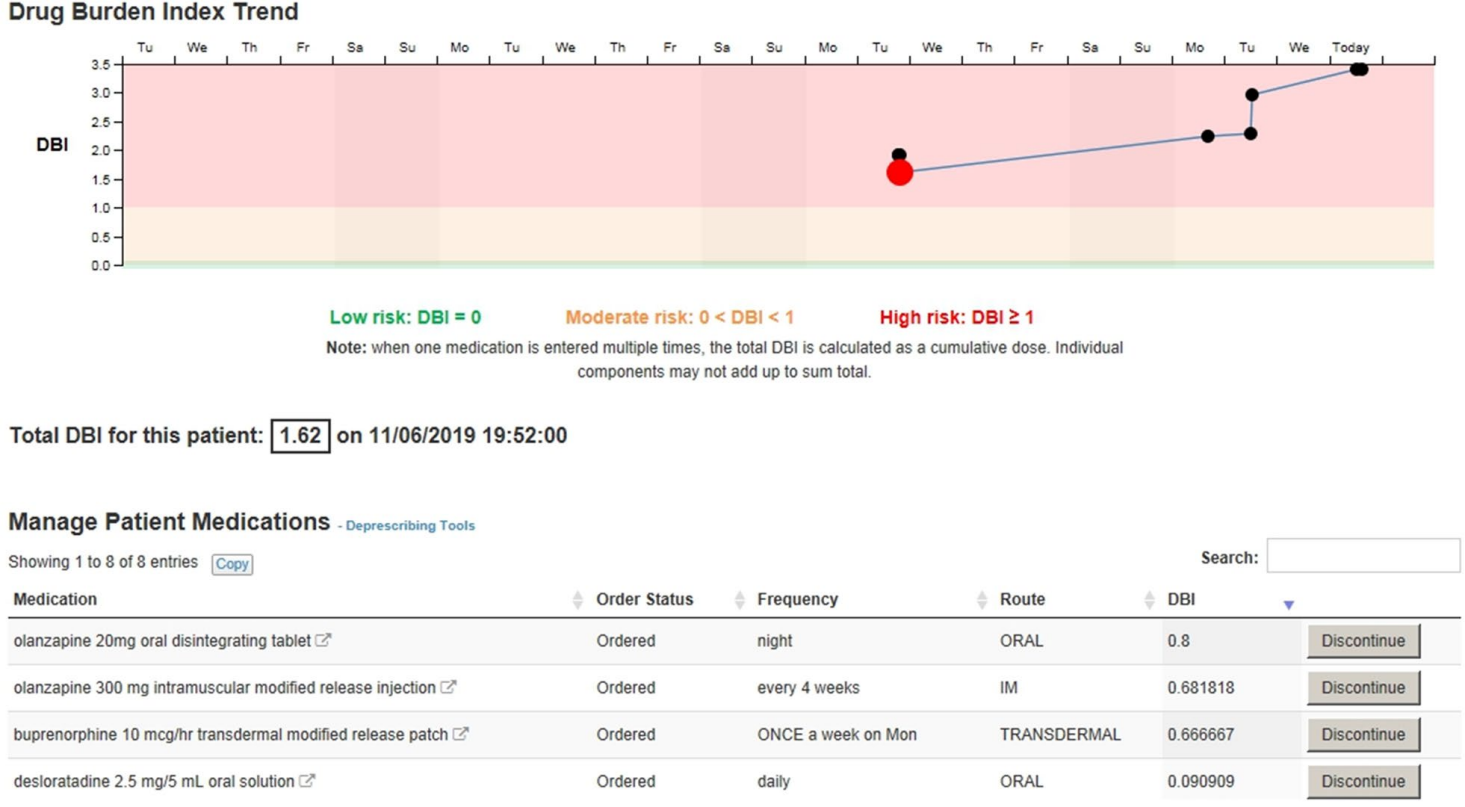
Screen shot of DBI MPage prior to usability testing

Our CDS tool comprised multiple components within the electronic medical record, informed by a large number of focus groups and interviews with end-users [6], including review of paper prototypes. Central to our CDS tool is the concept of a DBI. We used high DBI (DBI > 1) as a trigger to prompt medication review and consideration of deprescribing, prioritising review of medications with anticholinergic and sedative effects. DBI is a clinical risk assessment tool that can be calculated from the information in a patient’s electronic prescription, without need for additional clinical information. In this paper, we report results related to only one component of our CDS tool, a DBI MPage. The MPage, or clinical workspace, is the primary work area for reviewing a patient’s DBI score (current and past) and medications in the electronic medical record. The DBI MPage is one of a number of pages available within the electronic medical record (i.e. it is not a separate system or external application). As shown in Fig. 1, it includes (1) a graph showing the trend in the patient’s DBI over the course of the current admission; clicking on a point on the graph displays the DBI score and the patient’s active medications on that day, (2) the patient’s total DBI score, (3) a list of the patient’s medications, each with a DBI score, (4) hyperlinks to deprescribing guides and consumer information leaflets, (5) buttons beside each medication which direct the user to the orders page for deprescribing, and (6) a help function.

### Participants

In total, 17 individuals participated in the formative usability testing, six in Round 1 and 11 in Round 2. They comprised three senior doctors (consultants), five registrars, two junior doctors (1–3 years post-graduation) and seven pharmacists. All worked at the same teaching hospital in Sydney, Australia. To recruit participants, managers of the departments of aged care, general medicine and pharmacy were contacted by email and were asked to nominate participants, who were then contacted by a member of the research team and invited to take part.

### Testing scenarios

Ten scenarios were developed by a Professor of geriatric pharmacology (SH) and pharmacist researcher (MD), with the assistance of a Human Factors researcher (MB) and two eHealth experts (MSB and SA). Scenarios were designed to test all functionalities (i.e. use all parts of the DBI MPage) and possible outcomes of the tool. An outcome common to doctors and pharmacists was review of medications. For doctors, outcomes also included decision to deprescribe vs. not to deprescribe and initiating deprescribing vs not deprescribing in hospital or on discharge. For pharmacists, outcomes included providing advice to deprescribe vs. providing advice to not deprescribe. Example scenarios are presented in the Additional file 1: Appendix.

### Data collection and analysis

Testing was completed in a classroom on the campus of the hospital where the participants worked. At the beginning of the test session, each participant was briefed on the concept of a DBI, and on the purpose of the usability testing. Participants were then instructed to complete the scenarios while verbalising all thoughts as they interacted with the CDS tool. Two testers were present during each session, a pharmacist and an eHealth expert. One tester worked through scenarios with participants, prompting users to think aloud if they remained silent. The second tester acted as an observer. Following completion of scenarios, users participated in a brief semi-structured interview to explore their views of the CDS tool, including the component names, locations, navigation and utility. All test sessions, including semi-structured interviews, were audio-recorded and transcribed verbatim. The observer also took hand-written notes while observing scenarios.

All transcripts were reviewed and analysed independently by two Human Factors researchers (MB and WYZ), and observation notes were consulted if clarification was needed. Following independent analysis, the two researchers came together to discuss themes and reach a consensus on modifications or enhancements required for each CDS component based on user performance and feedback. These were presented to the wider research team, including members responsible for CDS redesign, to determine if modifications were feasible.

### Ethics approval

Ethics approval was obtained from Northern Sydney Local Health District Human Research Ethics Committee (LNR/17/HAWKE/138). All participants gave informed written consent to take part and to be audio-recorded.

## Results

### Usability issues identified during Round 1 testing

Participants expressed positive views about several components of the DBI MPage. For example, colour to indicate risk level was viewed positively: *“It’s obvious. So me, not being familiar with the numbers, it was easy for me to work out those probably are bad” (P2),* as was the complete medication list *“It’s nice just having a list of everyone’s medications because I find that…often a lot of our geriatric patients have a few, a lot of scrolling to do in order to get through all their medication. So, this is quite nice in the sense that it just gives you a list, which is very straightforward. And it just goes through what medications you would consider stopping”. (P7).*

However, participants also made a number of suggestions to improve the design of the page, primarily related to more clearly conveying the meaning of a DBI score and the DBI graph. Table 1 lists the usability issues identified, illustrative quotes, and recommended changes. The recommended changes in Table 1 were implemented prior to Round 2 testing.

**Table 1:** Issues uncovered during Round 1 testing, illustrative quotes and recommended changes to the CDS tool

Usability issue	Illustrative quote	Recommended change
Users were confused about what the DBI graph showed	I don't know what these dots are. So that was on the ninth, it was on the eighth and that was on the seventh… So the DBI is based on what they’ve charted as an inpatient? (P1)	Include a title or legend on the DBI graph, ensure “?” defines DBI and how it is calculated
Users were confused about the big red dot on the DBI graph	I don't know why there’s a big red dot. (P3)	Reduce current dot size, so all dots are the same size
Users reported the ‘Discontinue’ button was hard to see and were confused about the name of the button (e.g. do they click if only reducing a dose?)	If that had a drop down “modified dose” or “doctor dose reduction” or something, like a heading like that, because in my head you're not cancelling the drug completely. (P4)	Enlarge discontinue button and rename to ‘Modify’
Users reported that the link to deprescribing guides is too small and where the link is located makes it easy to miss	Let’s find the deprescribing tool. The font is too small, I think. It should be a little bit bigger. (P8)	Enlarge link to deprescribing guides and move to a more prominent position

### Usability issues identified during Round 2 testing

**Fig. 2:**
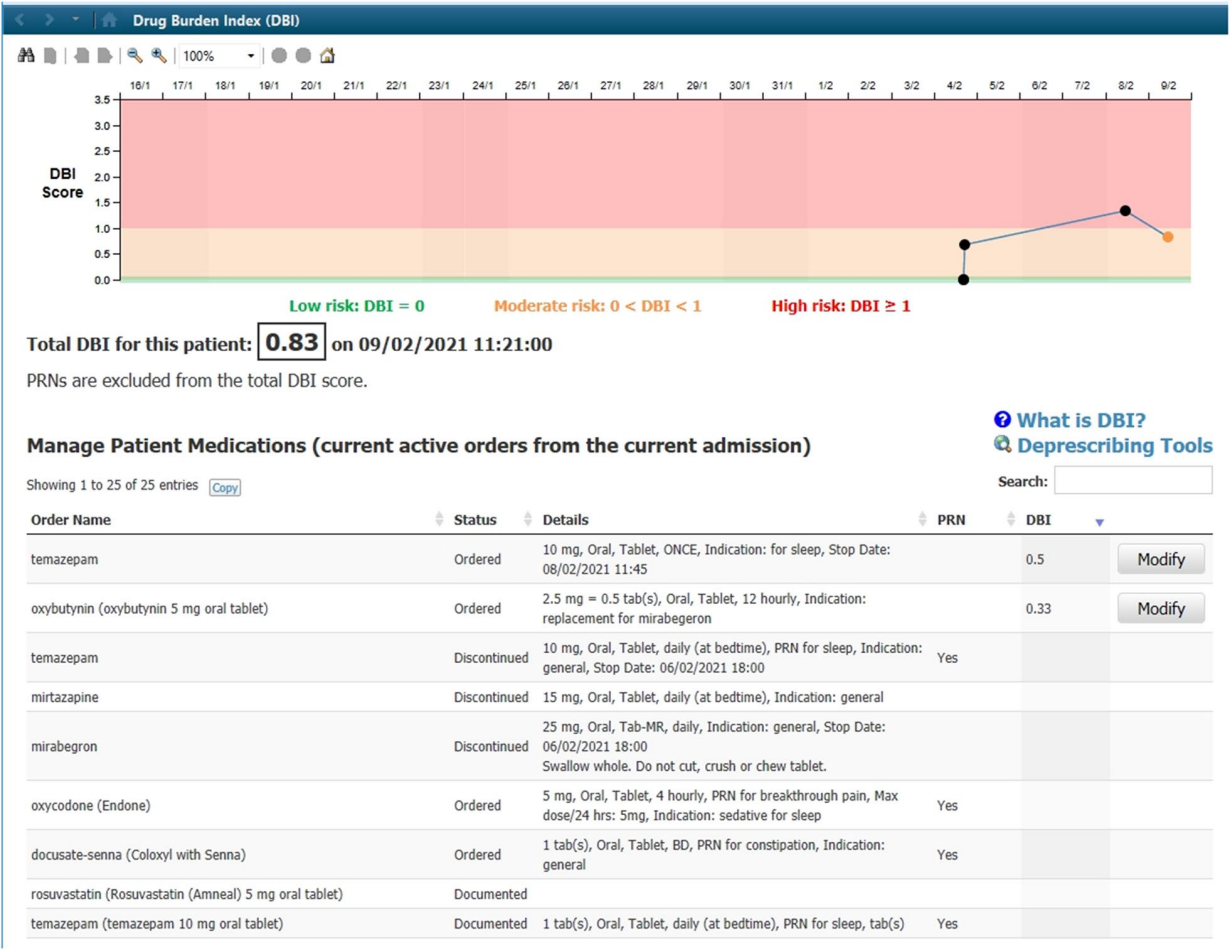
Screen shot of DBI MPage incorporating changes made following usability testing

Consistent with Round 1 testing, during Round 2, users continued to express positive views about the DBI MPage. For example, a participant said: *“It feels quite well-integrated into the current EMR (electronic medical record) system” (P6).* The deprescribing guides that were available via a hyperlink on the page were particularly valued. For example, a user said *“I’ll definitely use this. This looks amazing. I think that’s very useful” (P5).* Despite these positive statements, we identified a number of additional usability issues during Round 2 testing, as shown in Table 2. These largely related to consistency. Users wanted information and functionality on the DBI MPage to be consistent with other pages in the electronic medical record. Following Round 2 testing, additional modifications were made to the DBI MPage in response to the user feedback. An updated MPage appears in Fig. 2.

**Table 2:** Issues uncovered during Round 2 testing, illustrative quotes and recommended changes to the CDS tool

Usability issue	Illustrative quote	Recommended change
Users reported that the total DBI score on the MPage could be made more obvious, as this was lost among other information	The score here didn't really jump out in my eyes because of the graph is so more visually stimulating that I didn't actually realise that was there. (P10)	Increase font size of DBI total score
Users were confused about what medications were listed—were these only for the current admission?	Just to clarify, I know the status is on there, or are these drugs ordered on the drug chart currently? (P12)	Include a title above the medication list to indicate that these are current active orders from the current admission
When previous dots on the graph were clicked, it was not clear to users that the med list changed to reflect medication orders current on that day	I can see that if you click on the dots, you can actually see the DBI as the medications change. However, my first instinct was just to hover because in the “between the flags” section of the EMR you can just hover around the blood pressures and it will tell you what it is. (P6)	When previous dates are being viewed (i.e. previous dots are clicked), 1) Change the title on the medication list to indicate that this is a previous list (consider inserting date) and 2) Lighten medications (grey out) to make clear that these are not current medications
It was not clear to users that PRNs were not included in the DBI calculation and almost all users asked about this	It implies that these ones aren’t risky drugs at all … is there some place you could put a little note saying that it’s PRN and that’s why it’s not in the calculation? (P13)	Include a statement near the DBI total score to explain that PRNs are excluded
Users were confused about which dates were being displayed	We’re used to, with the results page or the obs(ervation) page, seeing the date across the top (P11)	Include dates on the DBI graph consistent with format of other graphs in EMR (e.g. “Between the flags” graph)
Users did not access the ‘?’ but asked about DBI, what it means and how it is calculated (for what patient ages, are stat medications included, based on orders or administrations?)	I think this little question mark, I think if you had like ‘What is the DBI?’ or something because I don't know if people would know to go and look up there for. (P12)	Include a ‘what is DBI’ hyperlink on the MPage which describes what DBI is and how it is calculated

*DBI* Drug Burden Index, *PRN* pro re nata (as needed)

## Discussion

Formative usability testing revealed a number of clear areas for improvement of our CDS tool, primarily related to terminology used, and consistency of functionality and display. Users were unfamiliar with the concept of a DBI score and suggested the inclusion of a definition and clear explanation of the DBI in the help section of the tool. Additional information was also required to allow accurate interpretation of the DBI graph. Furthermore, users expressed a preference for the CDS tool to look and function in a similar way to other decision support tools in the electronic medical record.

These findings are not unexpected, as they reflect two well-known usability heuristics: *match between system and the real world* and *consistency and standards *[30]. Systems should be designed to speak the user’s language, including words, phrases and concepts familiar to users. Systems should also be consistent, with all actions, functions and words meaning the same thing when used on multiple screens or locations. These principles are fundamental to good user-centred design [30]. Our results highlight the importance of consistency in both visual display and functionality when designing CDS tools in an electronic medical record.

Consistency is one of the greatest contributors to usability, with research showing that users will be faster to learn and use a new tool or system if it includes elements they are familiar with, or elements they frequently encounter while using other tools or systems [31]. Although consistency in design appears to be at odds with innovation, usability testing is an ideal way to determine whether a new display or feature, one that is inconsistent with other elements, will be understood and used by end-users [32]. In this study, we attempted to include a novel feature on our DBI graph: clicking on a point on the graph displayed medication data below the graph corresponding to that point in time. Users were confused by this feature, were not able to use it unprompted, and expressed a preference for having the graph function in a similar way to other graphs in the electronic system. Formative usability testing thus allowed us to determine this feature, in its current form, is unlikely to be used by clinicians. We recommend this approach for testing of any new CDS feature or function.

General impressions of the CDS tool were positive and we hope that our revisions to the tool based on this testing will result in a tool that is used and integrated into current clinician work processes. We appreciate that the CDS tool is unlikely to address all the barriers we identified in our preliminary work [6]. However, if used as intended, the tool directly addresses key barriers, namely fitting with the timeframe and workflow of an acute admission, and uncertainty and insecurity about deprescribing. If all design elements are used, the CDS tool will (1) make it possible to quickly identify patients with a high DBI who could be candidates for deprescribing, (2) facilitate review of a patient’s medications over the course of their admission, (3) provide access to guidance on whether and how to deprescribe, (4) provide clinicians with relevant information to pass onto consumers, and (5) allow easy navigation to the page in the electronic medical record where deprescribing is possible.

### Limitations

Participants were naïve not only to the CDS tool, but to the concept of a DBI, and this impacted their perception of the intervention. Real-life implementation of the CDS tool is likely to be accompanied by an education module on reviewing polypharmacy in hospitalized patients (https://www.heti.nsw.gov.au/education-and-training/my-health-learning; course code: 185346268), which includes information on DBI. Formative testing allowed us to identify a number of usability problems with the CDS tool, but represents only one component of usability evaluation, and we recommend additional testing be undertaken to assess efficiency, effectiveness, and satisfaction following integration of the CDS tool into clinical workflow.

## Conclusion

To ensure our CDS tool is used, usable and useful, we undertook usability testing and identified a number of ways to improve the layout, content and components of the tool. This approach proved extremely informative and we recommend formative usability testing be undertaken prior to implementation of all digital health interventions. Key lessons include ensuring CDS tools include visual displays and functionalities (i.e. a look and feel) consistent with other components in the eMR, and incorporating terminology known to end-users. We hope our revised CDS tool will address a key barrier to deprescribing by equipping clinicians with the knowledge and confidence to consider deprescribing in hospitalised patients within their limited timeframes and existing workflows. In the next phase of our project, we plan to test our CDS tool in practice in order to evaluate its uptake and effectiveness in hospital geriatric medicine and internal medicine settings.

## Supplementary Information

**Additional file 1**. Example usability testing scenarios for pharmacists, junior doctors, and registrars and senior doctors.

## Data Availability

The datasets used and analysed during the current study are available from the corresponding author on reasonable request.

## Abbreviations

EMR: Electronic medical record
DBI: Drug Burden Index
CDS: Computerised decision support
PRN: Pro re nata
