# Supplementary material for: Supporting deprescribing in hospitalised patients: formative usability testing of a computerised decision support tool
Source: BMC Med Inform Decis Mak. 2021 Apr 5;21:116. doi: 10.1186/s12911-021-01484-z (PMC8022373; doi:10.1186/s12911-021-01484-z)
Supplement: Supplementary file 1 — Additional file 1. Example usability testing scenarios for pharmacists, junior doctors, and registrars and senior doctors. [file 12911_2021_1484_MOESM1_ESM.docx]

**Supporting deprescribing in hospitalised patients: Formative usability testing of a computerised decision support tool**

Melissa T Baysari, Mai Duong, Patrick Hooper, Michaela Stockey-Bridge, Selvana Awad, Wu Yi Zheng, Sarah N Hilmer

**Appendix – Example usability testing scenarios**

**A.1. Scenario for pharmacists**

Patient name: Mrs Elinor Dashwood

Next Person of Kin name: Mr Edward Ferras (husband)

DOB: 18/02/1939

Doctor: Dr John Willoughby

**Medical Conditions:** Cognitive impairment, Hypertension, Depression, CCF, diabetes, OA, NSTEMI

**Medication list:**

| Diazepam 5mg – 1 nocte, PRN anxiety (but taken regularly) (DBI= 0.5) | Metformin 1000mg – 1 mane |
| --- | --- |
| Furosemide 40mg - 1 daily | Citalopram 40mg – 1 mane (DBI=0.8) |
| Perindopril 5mg - 1 daily | Diclofenac 50mg – 1 tds prn |
| Clopidogrel 75mg - 1 daily | Rosuvastatin 10mg – 1 mane |
| Bisoprolol 2.5 mg - 1 daily | Docusate and Senna - 2 nocte prn |
| Paracetamol 500mg\|codeine 8mg – 2 QID PRN |  |

**Key Findings:**

Mrs Dashwood is a 79-year-old female who is experiencing increased confusion. She walks with a rollator as she has a history of OA. She had a recent admission to the hospital due to CCF and was commenced on furosemide, perindopril and bisoprolol. She was commenced on diazepam several years ago for depression, around the time her son fell ill. Mr Ferras is concerned that Mrs Dashwood is always drowsy and sleeps throughout the day. Their overall goal is to optimise her quality of life. Her husband is concerned about the amount of medications she takes and that medications may be causing side effects.

Mrs Dashwood was admitted last night for a fall. You are reviewing Mrs Dashwood’s medications as a new admission with a high falls risk. You notice that she takes multiple medicines with high DBI scores. She is unstable, increasingly confused, and drowsy in the daytime. Her husband is worried about the number of medicines she’s taking and would like to optimise her quality of life. You identify that she started on diazepam a few years ago for depression when her son passed away due to illness. You wonder if the high dose of citalopram can be reduced. You think that the indication is inappropriate and would like to see her come off the diazepam*,* but unsure whether it should be deprescribed or how to do it.

**Task:**

1. *Starting from the Pharmacy Care Orangiser (PCO) patient list, select the patient Elinor Dashwood -Eight- MRN 544697.*
2. *Review Mrs Dashwood’s* *DBI Score.*
3. *Click on the Drug Burden Index (DBI) “score” to visit the Patient Level DBI MPage.*

You look up how this can be done and want to reduce the diazepam and citalopram, monitor and follow up with the GP after discharged home. You speak to the patient and their family who agree with this plan. You give the JMO a call to make a recommendation.

1. *Leave a note recommending diazepam to be reduced with aim of stopping.*
2. *Recommend discontinuing the citalopram.*
3. *Document this medication change.*
4. *Prepare and print Consumer Information Leaflets for benzodiazepams and z-drugs for Mrs Dashwood and her family.*

**A.2. Scenario for junior doctors**

Patient name: Mrs Jean Matthews

Carer name; Mr Jason Matthews (Son)

DOB: 18/02/1939

Address: 67 Piccolo Street, Smallville, NSW 2222

Date of Medication Review: 13/02/2017

Referring Practitioner: Dr Graham Norton

**Medical Conditions**: Hypertension, Depression, Diabetes, chronic back pain (>6years)

**Medication List:**

| Tapentadol SR 200mg - 1 bd | Metformin 1g - 1 mane |
| --- | --- |
| Telmisartan/hydrochlorothiazide 80/12.5mg - 1m | Citalopram 40mg - 1 m *(DBI=0.8)* |
| Temazepam 10mg - 1 nocte prn | Diclofenac 50mg - 1 tds |
| Tramadol 200mg - 1 bd *(DBI=0.67)* | Rosuvastatin 10 mg - 1 nocte |
| Codeine 30mg - 1 AID prn |  |

**Key findings:**

- Jason is very organized with his mom’s medications, however is concerned about the amount of medications she takes. He explains that she is always drowsy and ‘not herself’ as she always seems sedated.
- It is noted that Mrs Matthews has been prescribed 5 different types of painkillers and anxiolytics by several different doctors. To control her back pain, she switches between taking tapentadol and tramadol (thinking it is the same drug), and takes the strong painkiller with codeine regularly.
- She explains that she is constipated on most days and takes prune juice when she wants to relieve the constipation.
- Jason and Mrs Matthews would like to establish a better pain control for Mrs Matthews’ back pain. Their overall goal is to optimise functional independence.

You and the medical team visit Mrs Matthews on the daily ward round and find her son Jason waiting to speak about his concerns with his mother’s medications. You identify that Mrs Matthews seems confused and sedated. Her pain is not under control, but takes several pain medications and anxiolytics prescribed by different doctors. To control her back pain, she switches between taking tapentadol and tramadol (thinking it is the same drug), and takes the codeine regularly, and is suffering from constipation. Her son Jason would like to establish better pain control for Mrs Matthews’ back pain and optimize her functional independence. He would like to know what symptoms to look out for if she comes off any of the medications.

**Task:**

1. *Starting from the DBI Orangiser Level Patient List, select the patient Jean Matthews-One - MRN 544655.*
2. *Go to Patient’s Summary Page and review Mrs Matthew’s* *DBI Score.*
3. *Click on the Drug Burden Index (DBI) tab to visit the Patient Level DBI MPage.*
4. *Access the deprescribing tools from the Patient Level DBI MPage.*

You think tapentadol and temazepam may be inappropriate for this patient, but unsure whether it should be deprescribed or how to do it. You look up how to wean these medications and ask the registrar for their advice. The registrar agrees that reducing the dose of tapentadol and temazepam is appropriate, with referral to physio and psychiatrist to help pain management strategies and assess if citalopram can be reduced. The patient and their family agree with this plan.

1. *Return to the Patient Level DBI MPage and “Modify” the tapentadol from the Patient Level DBI MPage.*
2. *Prescribe a lower dose of tapentadol, ensuring you document the reason for discontinuing.*
3. *Document this medication.*
4. *Discontinue the temazepam.*
5. *Prescribe a lower dose of temazepam.*
6. *Document this medication change.*
7. *Document this medication change in the discharge summary documentation.*
8. *Prepare and print Consumer Information Leaflets for benzodiazepams and z-drugs for Mrs Matthews and her family.*

**A.3. Scenario for registrars and consultants**

Patient Name: Mr Fitzwilliam Darcy

Next person of Kin: Mrs Jane Austen

DOB: 12/01/1944

Referring Practitioner: Dr Amy Bennet

**Medical conditions**: Osteo-Arthritis, Osteoporosis, fracture in Left humerus, Recurrent falls, Depression, Pancytopenia

**Medication List:**

| Sertraline 100mg - 1 mane *(DBI=0.67)* | Temazepam 10mg - 1 nocte prn sleep (but taken regularly) *(DBI=0.5)* |
| --- | --- |
| Paracetamol 1000mg - 1 TDS PRN | Celecoxib 200mg -1 mane |
| Alendronate 70mg - 1 weekly on Mon | Esomeprazole 40mg - 1 daily |
| Calcium 600mg - 1 daily except Mon | Oxycodone 5mg - 1 tab 4 hourly prn pain |
| Vitamin D 1000IU daily | Docusate with Senna - 2 nocte prn |

Key Findings:

Mr. Darcy is an 88-year-old male who has difficulty sleeping and is taking Temazepam every night, which he says helps most of the time, but he’s drowsy most of the day and takes lots of naps. He has been admitted into hospital repeatedly due to falls. Mr. Darcy also reports that he experiences pain in the left hip and knee. During the Medication review interview, Mr Darcy reported to be taking paracetamol several times during the week, there is uncertainty that the oxycodone is providing relief from pain. Mr Darcy reported that his main goal is to optimise quality of life and to be pain free. Mr Darcy feels that he spends a lot of money on his medicines and his medicines are a weighty burden to him.

Mr Darcy was admitted again last night for a fall. You and the medical team visit him on the first ward round assessment. He takes multiple medicines with high DBI scores. His hip and knee pain is not well controlled and he is drowsy all day. He would like to reduce the number of medicines he’s on to save money. You would like to reduce sertraline and temazepam, but unsure whether it should be deprescribed or how to do it.

**Task:**

1. *Starting from the DBI Orangiser Level Patient List, select the patient Fitzwilliam Darcy-Four - MRN 544672.*
2. *Go to the Patient’s Summary Page, review Mr Darcy’s* *DBI Score.*
3. *Click on the Drug Burden Index (DBI) tab to visit the Patient Level DBI MPage.*
4. *Access the deprescribing tools from the Patient Level DBI MPage.*

You also think Mr Darcy’s pain should be reassessed and refer him to physio. You decide reducing the temazepam is appropriate but will refer to a psychiatrist to reassess the sertraline. The patient and their family agree with this plan.

1. *Return to the Patient Level DBI MPage and “Modify” the temazepam* *from the Patient Level DBI MPage.*
2. *Prescribe a lower dose of temazepam, ensuring you document the reason for discontinuing.*
3. *Document this medication change.*
4. *Document this medication change in the discharge summary documentation.*
5. *Prepare and print Consumer Information Leaflets for benzodiazepams and z-drugs for Mr Darcy and his family.*
